# GALNT2 sustains glioma stem cells by promoting CD44 expression

**DOI:** 10.18632/aging.204609

**Published:** 2023-03-25

**Authors:** Yingying Liu, Peisheng Chen, Liufei Xu, Bo Wang, Shengping Zhang, Xiangpeng Wang

**Affiliations:** 1 Department of Neurosurgery, First Affiliated Hospital of Kunming Medical University, Kunming, China

**Keywords:** glioma stem cell, CD44, GALNT2

## Abstract

Glioblastoma (GBM) is the most prevalent and malignant brain tumor and is highly resistant to currently available treatment. In this study, we reveal that polypeptide N-acetylgalactosaminyltransferase 5 (GALNT2) expression level was elevated in GBM, IDH1 wildtype glioma, and GBM stem cells (GSCs). GALNT2 increased expression correlated with GBM patients’ unfavorable clinical outcomes. Functionally, targeting GALNT2 blocks GSCs cell proliferation, self-renewal, and malignant invasion through repressing CD44 expression. Most importantly, we first provide evidence suggesting that STAT3 activates GALNT2 expression at the transcriptional level by directly binding to the GALNT2 promoter. Through a rational screening, we found a GALNT2 inhibitor that dramatically suppresses GSCs self-maintenance *in vitro* and *in vivo*. Collectively, we uncovered the critical function of GALNT2 in promoting GSCs self-maintenance and GBM progression and may provide a new potential drug for GBM clinical therapy.

## INTRODUCTION

Glioblastoma (GBM) is one of the most prevalent and lethal solid tumors with an abysmal prognosis [1]. Despite recent advances in treating GBM, including surgery, chemotherapy, and radiation, the median survival time of GBM patients remains at only 15 months [2, 3]. The worse prognosis and subsequent tumor relapse of GBM could be partially attributed to the tumorigenic potential of glioblastoma stem cells (GSCs). GSCs are a small subpopulation of cells enriched at GBM niches and are defined by having self-renewal ability and tumorigenic potential simultaneously, which account for tumor initiation, invasion, malignant development, therapeutic resistance, and tumor relapse [4–7]. Accordingly, targeting GSCs may open a new direction for GBM clinical therapy.

Aberrant activation of STAT3 is frequently observed in GBM and was found to be a requirement for GSCs stemness maintenance [8]. Briefly, the canonical STAT3 signaling is activated by the interleukin-6 (IL-6) cytokine family, followed by the dimerization of the IL-6 receptor glycoprotein 130 (gp130), which results in phosphorylation and activation of Janus family kinases (JAKs) [9]. Activated JAKs phosphorylate and activate STAT3 via inducing its nuclear translocation to transcriptional activation of downstream genes [10], which has been well-established to be essential for glioma initiation and tumorigenesis. However, the downstream target gene of STAT3 signaling is not fully understood. Thus, it is imperative to gain a deeper understanding of the mechanisms underlying aberrant activation of STAT3 signaling in GSCs, which may accelerate the progression of therapeutics to eliminate GSCs selectively.

CD44 is a non-kinase family and single-transmembrane glycoprotein that has been widely accepted as a requirement for cancer stem cell maintenance [11]. The expression of CD44 could be associated with tumor subtypes and be a biomarker of cancer stem cells [12], nonetheless, it is impossible to target CD44 directly for cancer therapy since CD44 is also required for the function of stem cells as well as immune cells [13]. Accordingly, targeting CD44 upstream regulators may provide a new option for cancer therapy.

The GALNT2 (polypeptide N-acetylgalactosaminyltransferase 2) is a member of the glycosyltransferase protein family, whose biological function and mechanism remain relatively unknown. GALNT2 displays contradictory functions in different types of tumors. According to recent research, GALNT2 inhibits the malignant functions of gastric cancer cells by blocking EGFR signaling [14]. Researchers reached contrary conclusions in lung adenocarcinoma [15], oral squamous cell carcinoma [16], and hepatocellular carcinoma [17]. However, the functional role and molecular mechanism of GALNT2 in GBM progression remain elusive. Thus, based on this background, we aimed to explore the biological significance of GALNT2 in GBM tumorigenesis and development.

## MATERIALS AND METHODS

### Clinical glioma specimens

In this study, all clinical glioma specimens were collected from the First Affiliated Hospital of Kunming Medical University. This research was authorized by the Institutional Ethics Committees of the First Affiliated Hospital of Kunming Medical University.

### Antibodies and reagents

For western blotting in this study, the following primary antibodies were used: b-actin (Ab8226; Abcam) and rabbit control IgG (AC011; Abclonal). GALNT2 (ab140637; Abcam). STAT3 (12640; Cell signaling technology), CD44 (Ab24389; Abcam). STAT3 inhibitor GSK343 (HY-13500) and the FDA-approved drug library (HY-L022M) were purchased from MedChemExpress, Shanghai, China. Glioma stem cells and their corresponding non-glioma stem cells were isolated and maintained as per our previous study [18].

### Construction of stable cell lines

For cell transfection, negative targeting control (shNC) or STAT3 shRNA plasmids were co-transfected into HEK293T with pMD2.G and psPAX2. The lentivirus was collected after 48 h post cell transfection and infected indicated GSCs for 8 h and selected by puromycin (1 μg/mL) for around 7 days. The sequences of shRNA are listed in Table 1. To establish GALNT knock-out cell lines, sgRNA guides (sequences as listed in Table 1) were ligated into Lenti-multi CRISPR (Addgene 85402) plasmids. GSCs were transfected with 3 mg CRISPR-Cas9 knockout plasmids using Lipofectamine 3000 to isolate a single colony. After 48 h of GALNT2 knock out plasmid transfection, cells were treated with 1 μg/ml puromycin for 7 days. Single cells were isolated into 96-well plates. The knock-out colonies were validated by western blot analysis.

**Table 1:** Primer sequences used in this study.

**PCR primers**	
**Gene**	**sequence**
β-Actin-F	5’-GGGAAATCGTGCGTGACATTAAG-3’
β-Actin -R	5’-TGTGTTGGCGTACAGGTCTTTG-3’
GALNT2-F	5’-ACCAGGTGGAGAGTGATAAGC-3’
GALNT2-R	5’-TCCTCAGGATCATTGCTGTAGTC-3’
**shRNA or guide RNA**	**sequence**
GALNT2 guide RNA-#1	5’-ACGGTGGCCAGACTTTAACC-3’
GALNT2 guide RNA-#2	5’-TCTTTTTAGGGAAAGTACGG -3’
sh-STAT3	5’-CAGCTCTACAGTGACAGCTTC-3’

### Quantitative real-time PCR

Real time PCR assay was performed according to our previous study [19]. Briefly, total RNA was isolated from indicated GSCs according to the manufacturer’s guidelines. Reverse transcription was conducted using MuLV reverse transcriptase (2690S, Takara, Beijing, China) and Oligo(dT) primer. Quantitative real-time PCR was performed using SYBR Green PCR Mix (640022, Takara). β-actin was used as the internal control. The sequences of primers used in this study are listed in Table 1.

### Dual-luciferase assay

The luciferase reporter assay was performed according to our previous study [20]. Briefly, pGL4.2-GALNT2 3 kb promoter plasmids and the internal control plasmid pRL-TK were transfected into GSC1 which are seeded in 24-well plates up to approximately 60% confluence. Moreover, the STAT3 expression plasmid or empty vector was co-transfected for 48 h, and luciferase reporter activity was assayed using the Dual-Luciferase Assay System (E1910, Promega, USA) according to the manufacturer’s instructions.

### Tumor oncosphere formation and *in vitro* limiting dilution assay

Tumor oncosphere formation assay was performed as previously reported [18]. Briefly, single-cell suspensions were seeded into 6-well ultralow-attachment plates (3471, Corning, USA) at a density of 20, 000 cells per well using serum-free DMEM/F12 (Hyclone) containing 20 ng/mL of basic fibroblast growth factor, 20 ng/mL of epidermal growth factor, and 2 mM L-glutamine. After 10 days of maintenance, the number of tumor oncospheres was counted under a microscope. For *in vitro* limiting dilution assay, dissociated single GSCs were seeded in ultra-low attachment 96-well plates at the density of 1, 5, 10, and 20 cells per well using an oncosphere culture medium. After 10 days of culture, oncospheres in each well were recorded, and oncosphere formation frequency was calculated using online *in vitro* limiting dilution analysis (http://bioinf.wehi.edu.au/software/elda/).

### EdU-DNA synthesis and cell proliferation assay

The assay was performed according to instructions from EdU Cell Proliferation Kit (C10337, Invitrogen, USA). Briefly, indicated GSC1 were seeded at a density of 6×10^4^ per well into 48-well plates. After 24 h culture, 50 μM EdU solution was diluted in the cell culture medium, followed by adding into indicated GSC cells and then incubated at 37° C for another 2 h. The BrdU positive ratio determined by imageJ. Cell proliferation assay was performed as previously before [21]. The significance of difference was determined by the last day of the experiments.

### Western blot

GBM cells were washed in PBS and lysed with ice-cold RIPA buffer (Beyotime, P0013B, China) containing with protease inhibitor (Beyotime, P1010, China). The same protein quality was loaded to SDS-PAGE and transferred to a polyvinylidene difluoride membrane. Membranes were immunoblotted with specific antibodies according to standard protocols followed by blocking with 5% milk.

### Trans-well

Cell migration ability was detected using 8 μm trans-well inserts (Corning, USA). Briefly, a total of 3,000 GSC1 and GSC2 GALNT2 knockout or non-targeting control cells were resuspended in 150 μL serum-free culture medium and plated into the upper chambers, and the lower compartment was filled with 700 μL medium containing 20% FBS. After they were cultured 36 h at 37° C, non-migrating cells on the upper chambers were removed by cotton swab, and the cells adhering to the bottom side of inserts were fixed with 4% formalin for 20 min, and then stained with 0.2% crystal violet for 30 min. After they were rinsed with PBS, migrating cells were imaged by the microscope, and recorded.

### Immunofluorescence

For the immunofluorescence staining, glioma specimens were deparaffinized and rehydrated through an alcohol series, and followed by staining with primary antibodies against GALNT2 and CD44 using standard procedures. We quantified the score of GALNT2 and CD44 expression as immunohistochemistry quantification as described in immunohistochemistry.

### Chromatin immunoprecipitation assay

Ch-IP (Chromatin Immuno-Precipitation) assay was performed in GSC1 cells using Ch-IP kit (Millipore, USA) according to manufacturer’s instructions. Briefly, after cross-linking, cells were lysed and sonicated (20 seconds for 3 times). Sonicated lysates were then centrifuged at 14,000 rpm at 4° C for 20 minutes and the cleared chromatin (100 mg) was immunoprecipitated with 5 mg of anti-STAT3 antibody and incubated overnight at 4° C with gentle rotation. Quantitative PCR was performed for the detection of the bind site.

### Flow cytometry

Flow cytometry was performed according to our previous study [19].

### *In vivo* xenograft tumor mice model

Xenograft tumor model was created according to the previous study [22]. Briefly, a total of 1 × 10^6^ indicated GSC1 cells were diluted into 3 mL PBS and then implanted into the frontal lobes of 6-week-old female NOD/NSG mice. NOD/NSG mice were purchased from Beijing Vital River Laboratory Animal Technology Co., Ltd (Beijing, China). All animal studies were approved by the Animal Ethics Committee of Kunming Medical University.

### Immunohistochemistry (IHC)

Immunohistochemistry was performed according to our previous study [21]. Briefly, tissue slides were subjected to deparaffinize, rehydrate, and antigen retrieval. The tissue slides were subsequently incubated with primary GALNT2 antibodies overnight at 4° C. After carefully washing three times, horseradish peroxidase (HRP) conjugated secondary antibody was used to incubate the slides before DAB detection. For the IHC and immunofluorescence results analysis, the percentage of positive cells and staining intensity were scored as follows: 1 indicated that 0-25% of the tumor cells showed positive staining, 2 indicated 26-50% of cells were stained, 3 indicated 51-75% stained, and 4 indicated 76-100% stained.

### Bioinformatic analysis

To comprehensively understand the expression profile of GALNT2 and its prognostic values in gliomas, transcriptome sequencing data and their corresponding clinical data of TCGA, CGGA and Rembrandt data sets were downloaded from http://gliovis.bioinfo.cnio.es/.

### Statistical analysis

All data were analyzed by GraphPad prism 9.0 and are shown as the mean ± SD. Student’s t-test was used in this study. Survival analysis was evaluated using the Kaplan-Meier method and assessed using the log-rank test. Statistical significance was acknowledged when p <0.05.

## RESULTS

### Clinical significance of GALNT2 in glioma

**Figure 1:**
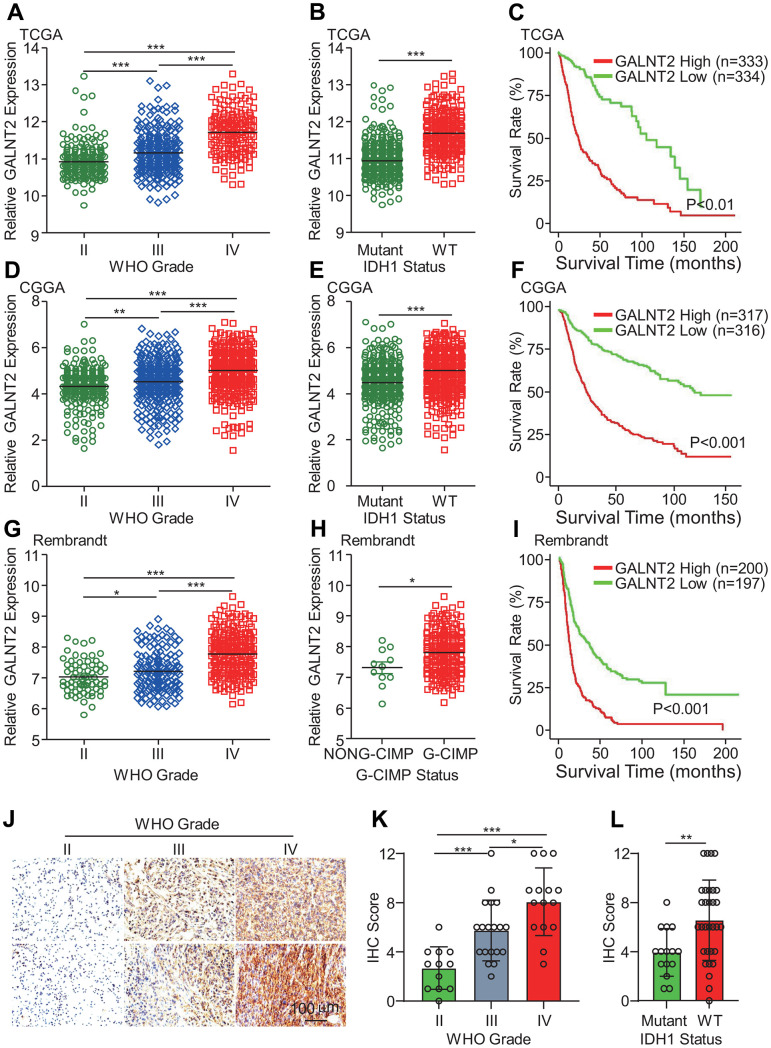
**GALNT2 expression was highly expressed in GBM and IDH1 wildtype GBM.** (**A**, **D**, **G**) GALNT2 expression was increased in WHO IV glioma in TCGA (**A**), CGGA (**D**), and Rembrandt (**G**) data sets. (**B**, **E**, **H**) GALNT2 expression was markedly elevated in IDH-wildtype gliomas in TCGA (**B**) and CGGA (**E**) and Rembrandt (**H**) data sets. (**C**, **F**, **I**) Increased GALNT2 expression predicts a poor prognosis of glioma patients assessed by Kaplan-Meier curves in TCGA (**C**) and CGGA (**F**), and Rembrandt (**I**) data sets. (**J**) GALNT2 protein expression was convincingly increased in WHO IV glioma. Representative images of IHC staining of GALNT2 protein in different grades of glioma specimens were shown. Scale bar: 100 μm. (**K**) Correlation between GALNT2 protein expression and WHO grades of gliomas. Clinical glioma specimens from 46 glioma patients were IHC-stained with anti-GALNT2 primary antibody. (**L**) GALNT2 expression was elevated in IDH1 wildtype group glioma patients. *, ** and *** indicate p < 0.05, p < 0.01 and p < 0.001, respectively.

To decode the expression profile and the potential clinical implications of GALNT2 in glioma, we analyzed RNA-sequencing data of glioma from the TCGA database. Compared with low-grade glioma patients (WHO grade II and grade III), GBM patients (WHO IV) group exhibited the highest GALNT2 mRNA expression, and GALNT2 mRNA expression level was highly related to patients’ grades (Figure 1A). Isocitrate dehydrogenase 1 (IDH1) mutation is commonly shown in about 80% of low-grade gliomas, and patients with IDH1 mutation frequently have a favorable prognosis, thus IDH1 mutational status is a strong prognostic biomarker for gliomas [23]. As shown in Figure 1B, GALNT2 mRNA expression was abnormally increased in IDH1 wild-type gliomas compared with the mutated gliomas. Additionally, there was a significant correlation between elevated GALNT2 expression in glioma patients and overall poor survival (Figure 1C). In line with this, almost similar results were found in the CGGA RNA-seq database (Figure 1D–1F) and Rembrant database (Figure 1G, 1I). Notably, glioma CpG island methylator phenotype (G-CIMP) is another prognostic biomarker of glioma patients, and patients with G-CIMP show worse clinical outcomes. Consistently, we found that GALNT2 mRNA expression was also elevated in patients with G-CIMP (Figure 1H). To further explore the clinical significance of GALNT2 in glioma, immunohistochemical staining was employed to characterize the *in vivo* expression of GALNT2 in different grades of clinical glioma specimens, and GALNT2 was found to be overexpressed in GBM than gliomas (Figure 1J, 1K). Additionally, GALNT2 expression was higher in IDH1 wild-type patients (Figure 1L). Thus, these results support that GALNT2 expression was a sign of unfavorable malignancy of gliomas and predicts poor survival of glioma patients.

### STAT3 binds to GALNT2 promoter and activates GALNT2 expression

**Figure 2:**
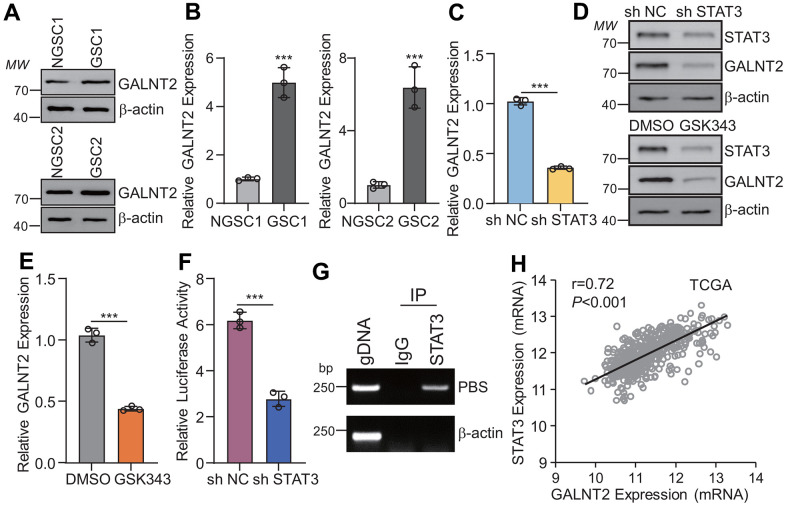
**GALNT2 is a downstream gene of STAT3.** (**A**, **B**) Both GALNT2 mRNA (**A**) and protein (**B**) levels were upregulated in GSCs as compared with their corresponding non-GSC cells. (**C**, **D**) Silencing STAT3 dramatically inhibits GALNT2 mRNA (**C**) and protein (**D**) expression. sh NC means non targeting control shRNA, sh STAT3 indicates STAT3 shRNA. (**D**, **E**) GALNT2 mRNA (**E**) and protein (**D**) expression was inhibited by treated GSC1 cells with 5 mM GSK343 for 48 h. (**F**) Luciferase construct containing full-length of GALNT2 promoter and STAT3 overexpression plasmid was co-transfected into GSC1 cells for 48 h. Transcriptional activation of STAT3 was determined by dual-luciferase reporter assay. (**G**) STAT3 directly binds to the GALNT2 promoter. (**H**) GALNT2 mRNA was positively correlated with STAT3 mRNA in the TCGA database. MW indicates molecular weight. *** p < 0.001.

To further investigate the functional role of GALNT2 in sustaining GSCs self-maintenance, patient-derived glioma stem cells (GSCs) and their corresponding non GSCs were isolated in GBM patients’ specimens, and it was observed that GALNT2 was significantly increased in GSCs compared with non GSCs (Figure 2A, 2B). To further determine the underlying mechanism of GALNT2 overexpression in GBM and GSCs, bioinformatics analysis was conducted to predict the potential transcription factors that transcriptionally activate GALNT2 expression, and it was observed that there are four potential DNA binding sites for STAT3 binding in the JASPAR database (http://jaspar.binf.ku.dk/) (data not shown). To this end, we established cell lines that stably knockout STAT3 in GSC1 and GSC2 cells, and we observed that silencing STAT3, but not STAT1 or STAT5 (data not shown), dramatically suppresses both GALNT2 mRNA and protein expression (Figure 2C, 2D). In line with this, GSCs treated with STAT3 inhibitor GSK343 dramatically inhibits both GALNT2 mRNA and protein expression (Figure 2D, 2E). Furthermore, luciferase reporter vectors containing full-length GALNT2 or IL-6 promoters of well-known STAT3 target genes were used as a positive control. As expected, dramatically increased luciferase activity of GALNT2 or IL-6 promoter (data not shown) compared with empty control was observed (Figure 2F). Chromatin Immunoprecipitation (Ch-IP) assay was also employed to validate the observation that STAT3 transcriptionally activates GALNT2 expression and we observed that STAT3 binds to GALNT2 promoter (Figure 2G). Consistently, GALNT2 mRNA was all positively correlated with STAT3 mRNA or CD274 mRNA in the TCGA (Figure 2H), CGGA, and Rembrandt data sets (data not shown). Taken together, these data reveal that STAT3 binds to the GALNT2 promoter and transcriptionally activates GALNT2 expression.

### GALNT2 is required for GSCs cell self-renewal and invasion

**Figure 3:**
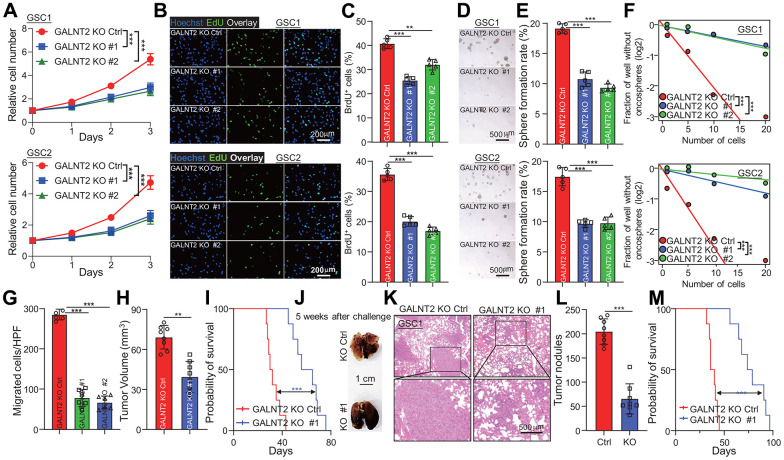
**GALNT2 is essential for GSCs malignant behaviors.** (**A**–**C**) Targeting GALNT2 clearly reduced indicated GSCs cell proliferation rate. As shown by cell proliferation assay (**A**, **B**) and EdU staining assay (**C**). Scale bar: 200 μm. (**D**) Depletion of GALNT2 markedly suppressed indicated GSC cells tumor oncosphere formation ability. Scale bar: 500 μm. (**E**) Quantification data for (**D**). (**F**) Indicated GSC cells tumor oncospheres formation ability was markedly blocked by depletion of GALNT2 determined by *in vitro* limiting dilution assay. (**G**) A total of 1.5 × 10^5^ GSC1 cells with or without depletion of GALNT2 were intracranially injected into NOD/NSG mice. Tumor growth was examined 5 weeks after the challenge, and representative H&E-stained coronal brain sections with tumor xenografts are shown. (**H**) Tumor volume was calculated. n=8 mice per group. (**I**) A total of 1.5 × 10^5^ GSC1 cells with or without depletion of GALNT2 were intracranially injected into NOD/NSG mice. Mouse survival time was recorded and presented with Kaplan-Meier survival curves. n=8 mice per group. (**J**) Images of lung of mice challenged with GSC1 cells expressed with or without GALNT2 knock out plasmid through intravenous injection. Mice were sacrificed at day 36 after intravenous injection of 1.5 × 10^5^ GSC1 cells, and the lung of mice was taken out to count tumor nodules. Scale bar: 1 cm. (**K**) Representative images of H&E-stained lung sections with tumor xenografts are shown. (**L**) Quantification of tumor nodules on NOD/NSG mice administrated with GSC1 cells expressed with or without GALNT2 knock-out plasmid. n= 8 per group. (**M**) Survival of NOD/NSG mice injected with GALNT2 knock-out or control cells after intravenous injection of 1.5 × 10^5^ GSC1 cells. ** and *** indicate p < 0.01 and p < 0.001, respectively.

To gain an insight into the biological functions of GALNT2 in GBM initiation and development, we knocked out GALNT2 in GSC1 and GSC2 cells. GALNT2 silencing markedly repressed GSCs proliferation, as reflected by reduced cell proliferation rate upon depletion of GALNT2 determined by detecting cell proliferation and EdU positive cells (Figure 3A–3C). Next, tumor oncosphere formation assay, a standard golden technique for detecting cancer stem cell self-renewal, was used to evaluate the potential effect of GALNT2 expression on sustaining GSCs self-renewal. In line with previous observations, tumor oncosphere formation ability of GSCs was significantly restrained following GALNT2 depletion (Figure 3D, 3E). This observation was further confirmed by cell proliferation and *in vitro* limiting dilution assay (Figure 3F). We next explore the role of GALNT2 expression in glioma tumorigenesis and malignant invasion. We observed that depletion of GALNT2 significantly decreased GSC migration ability determined by trans-well assay (Supplementary Figure 1A, 1B). GSC1 cells with or without depletion of GALNT2 were intracranially injected into NOD/NSG mice. As expected, silencing GALNT2 dramatically restricts tumor growth and augments the survival of mice. Additionally, we intravenously injected GSC1 cells expressed with or without depletion of GALNT2 into NOD/NSG mice (Figure 3H, 3I). We found that GALNT2 deficiency resulted in fewer lung metastases (Figure 3J–3L) and improved the overall survival of the mice (Figure 3M). Together, these results support that GALNT2 acts as an oncogene in boosting GSCs malignant behavior.

### GALNT2 promotes GBM tumorigenesis through promoting CD44 expression

**Figure 4:**
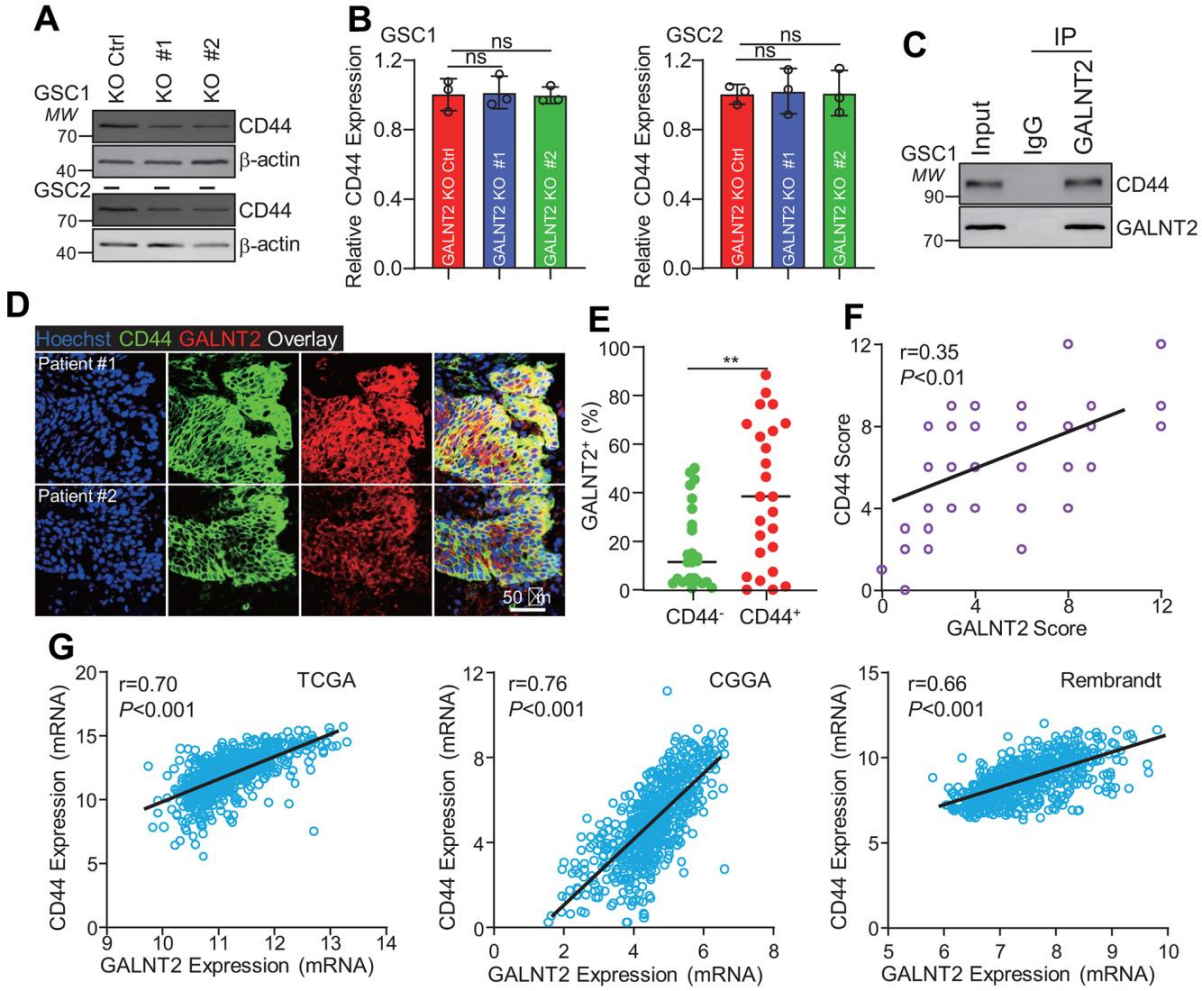
**GALNT2 promotes CD44 expression.** (**A**) Silencing GALNT2 significantly inhibits CD44 protein expression. (**B**) Silencing GALNT2 did not alter CD44 mRNA expression. ns indicates non significance. (**C**) GALNT2 interacts with CD44. IgG was used as a negative control. (**D**) GALNT2 co-localized with CD44 in GBM clinical specimens. Scale bar: 50 μm. (**E**) GALNT2 was preferentially expressed in CD44 positive cells. (**F**) GALNT2 protein expression was highly correlated with CD44 expression in GBM specimens. (**G**) GALNT2 mRNA expression was highly co-related with CD44 mRNA expression in TCGA (left panel) and CGGA (middle panel), and Rembrandt (right panel). ** indicates p < 0.01.

To further understand the underlying mechanisms that GALNT2 facilitates GBM development, several key proteins, including CD44, Sox2, Oct4, and ALDH that mediate the promotion of GSCs self-renewal and GBM progression were detected after silencing GALNT2 and found that only CD44 expression was considerably inhibited upon silencing GALNT2 (Figure 4A and data not shown). Intriguingly, we did not observe any expression changes of CD44 mRNA between GALNT2 knock out and control group (Figure 4B), suggesting that GALNT2 may regulate CD44 expression at the transcriptional level. Given that GALNT2 encodes a glycosyltransferase, and the enzymatic activity and candidate substrate were poorly understood, we wondered whether GALNT2 promotes GSCs self-renewal through directly interacting with CD44. To test this hypothesis, endogenous immunoprecipitation was performed, and GALNT2 was found to interact with CD44 (Figure 4C). This observation was further validated by co-immunofluorescence staining of CD44 and GALNT2 in GBM specimens (Figure 4D). Interestingly, we found that GALNT2 was preferentially expressed in CD44 positive GBM cells (Figure 4E). In addition, GALNT2 expression was highly co-related with CD44 in GBM specimens (Figure 4F). Furthermore, we found that GALNT2 mRNA expression was also co-related with CD44 mRNA expression in TCGA, CGGA and Rembrandt dataset (Figure 4G). Taken together, these results support that GALNT2 facilitates GBM progression by promoting CD44 expression.

### Rational screening GALNT2 specific inhibitor

**Figure 5:**
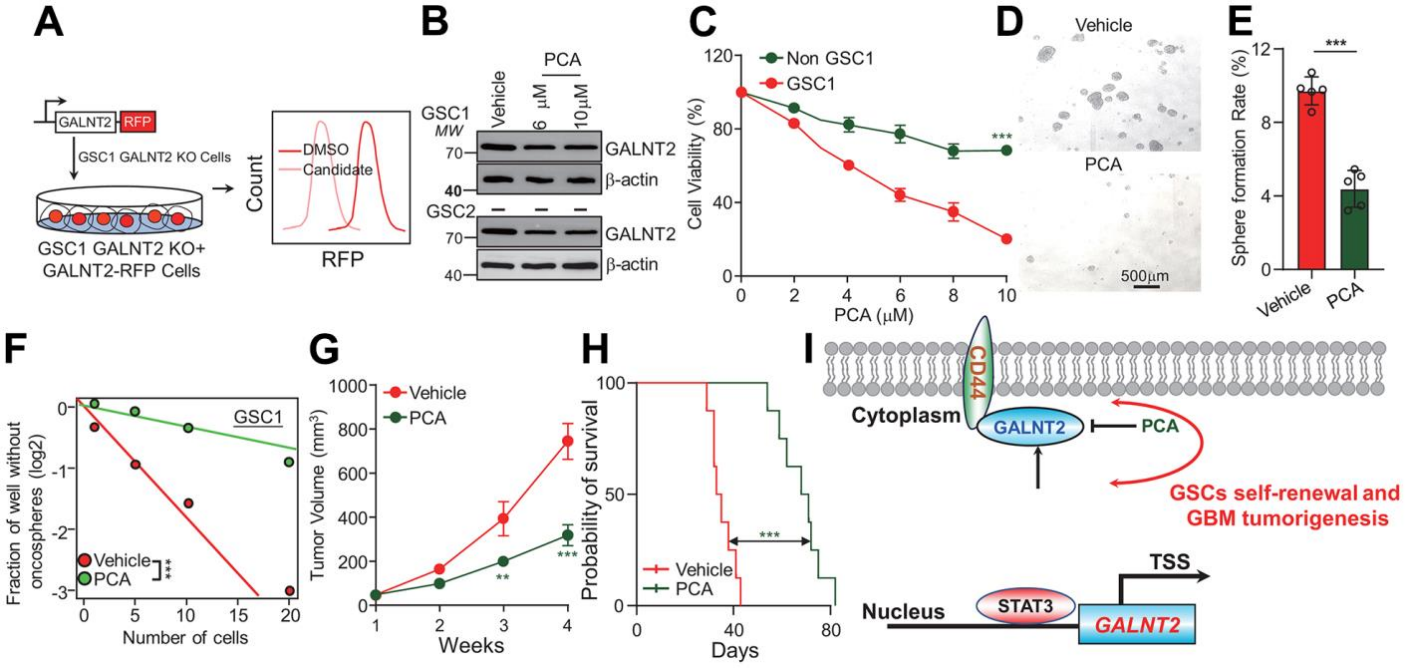
**PCA inhibits glioma progression by suppressing GALNT2 expression.** (**A**) Schematic illustration of GALNT2 inhibitor screening. RFP tagged GALNT2 plasmid was introduced into GSC1 GALNT2 knock out cells to establish GALNT2 re-expression cells. GSC1-GALNT2-RFP cells were treated with 10 μM of each FDA-approved drug candidate for 48 h, and then those cells were subjected to flow cytometry to screen GALNT2-specific inhibitors according to RFP intensity. (**B**) PCA treatment inhibits GALNT2 expression in GSC. (**C**) PCA treatment preferentially eliminates GSC, but does not impair non-GSC. (**D**) PCA treatment inhibits GSC oncosphere formation rate. Scale bar: 500 μm. GSC1 cells were treated with 6 mM PCA or DMSO for 48 h. (**E**) Quantification data of (**D**). (**F**) PCA treatment inhibits GSC self-renewal determined by *in vitro* limiting dilution assay. GSC1 cells were treated with 6 mM PCA or DMSO for 48 h. (**G**) PCA treatment suppresses GBM patient derived xenograft growth. Mice were intravenously injected with 10 mg/kg PCA or 5 % DMSO after 2 weeks of GSC1 cells were administrated into mice. n=5. (**H**) A total of 1.5 × 10^5^ GSC1 cells with or without depletion of GALNT2 were intracranially injected into NOD/NSG mice. Mice were injected with or without 10 mg/kg PCA every day for 8 days after administration of GSC1 cells *in situ*. Mice’s survival time was recorded and presented with Kaplan-Meier survival curves. (**I**) Working model of GALNT2 in sustaining GSCs. STAT3 transcriptionally activates GALNT2 expression in glioma stem cells, which then binds to and stabilizes CD44 and leads to glioma malignant development. TSS indicates transcription start site. n=8. *** p < 0.001.

Considering the critical role that GALNT2 plays in promoting GBM progression and the downstream signaling pathway in GBM, we aimed to develop a specific inhibitor of GALNT2 and translate it for clinical use. First, we established a stable cell line that re-expressed GALNT2 in GSC1 cells by introducing GALNT2 fused with a red fluorescent protein (RFP) into GSC1 GALNT2 knock-out cells, and screened a library of FDA-approved drugs using flow cytometry for evaluating RFP fluorescent intensity following treated with each FDA-approved drug (Figure 5A). Our lead compound was Procainamide (PCA). We further validate the flow cytometry screening results through immunoblotting. As shown in (Figure 5B), PCA treatment significantly inhibited GALNT2 expression. In addition, PCA treatment dramatically suppressed GSC, but not none-GSC, cell viability (Figure 5C). GSC1 self-renewal was repressed, followed by treatment with PCA determined by tumor oncosphere formation assay and *in vitro* limiting dilution assay (Figure 5D–5F). These observations suggested the inhibitory effect of PCA on GSCs stemness, which was consistent with the results in GALNT2 knock out cells (Figure 3). To further explore the effect of PCA on GBM *in vivo*, we administered PCA to GSC1 GBM xenograft growth, and found that PCA treatment significantly restricts GSC1 developed xenograft tumor growth and promotes the survival of mice *in situ* injected with GSC1 cells (Figure 5G, 5H). Importantly, administration of PCA did not alter the body weight of mice relative to the mice injected vehicle control. Additionally, Hematoxylin-eosin (H&E) staining showing that administration of PCA did not induce significant liver damage, suggesting that PCA did not exert a significant effect on general animal health (Supplementary Figure 2A, 2B). These results provide further confirmation that PCA holds great potential to function as a chemotherapeutic option for GBM patients.

## DISCUSSION

GBM is acknowledged as the most aggressive primary brain tumor, with a median survival time of 14 months [24, 25]. GBM recurrence is commonly inevitable, highlighting an urgent need to discover more durable treatment options for GBM. Glioma stem cell (GSCs) were considered to account for tumor invasion, progression, resistance to conventional therapy, and tumor recurrence [26]. Thus, it is of great significance to fully understand the molecular principles of GSCs self-renewal maintenance. STAT3 signaling was found to be commonly activated in GBM, and abnormal activation of STAT3 is essential for sustaining the self-renewal and tumorigenic potential of GSCs [8, 27–29]. However, the tumorigenic effects of STAT3 and the downstream genes of STAT3 are not fully understood. Since STAT3 signaling is also necessary for maintaining normal stem cell self-renewal and immune cell activation, direct targeting of STAT3 itself is not clinically feasible; however, targeting STAT3 downstream genes presents a new alternative for eliminating GSCs to improve GBM treatment.

In this study, we first defined that GALNT2 expression was high in GBM, GSCs, and IDH1 wildtype gliomas, and elevated GALNT2 expression was also associated with unfavorable GBM patients’ clinical outcomes. Mechanistically, we provide biochemical evidence that STAT3 binds to GALNT2 promoter and transcriptionally activates GALNT2 expression. GALNT2 promotes CD44 expression, which results in sustaining and development of GBM. Thus, GALNT2 functions as a critical molecular driver for GBM development. The genetic or pharmacological targeting of GALNT2 potentially inhibited the proliferation, invasion, formation of oncospheres, and growth of GBM cells (Figure 5I). An important limitation of this study is the lack of illustration for the underlying mechanisms by which GALNT2 facilitates GBM development. We therefore intend to extend our study by decoding how GALNT2 facilitates CD44 expression.

To date, many inhibitors have been reported to inhibit tumor progression in various cancer types, whereas only a small number of inhibitors have successfully entered into clinical trials and temozolomide is the only available clinically chemotherapy drug used in glioma treatment [28, 30–33], highlighting that there is an urgent need to characterize more inhibitors from FDA-approved drugs library that specifically target crucial oncoproteins and translate them into clinical therapeutics. Procainamide (PCA) is a medication of the antiarrhythmic class used to treat cardiac arrhythmias [34], but the potential effects of PCA in treating other diseases are largely unknown. We found that PCA treatment dramatically inhibits mice xenograft tumor growth and augments mice survival.

Collectively, our study presents findings on the crucial functions of STAT3/GALNT2/CD44 signaling axis in sustaining GSCs tumorigenic potential and the underlying molecular mechanisms. The high expression of GALNT2 in GBM holds great potential to translate into prognostic and diagnostic biomarkers. More importantly, GALNT2 inhibitor, PCA, may offer another therapeutic option for GBM patients’ therapy.

## Supplementary Material

Supplementary Figures
