# Supplementary material for: GALNT2 sustains glioma stem cells by promoting CD44 expression
Source: Aging (Albany NY). 2023 Mar 25;15(6):2208–20. doi: 10.18632/aging.204609 (PMC10085609; doi:10.18632/aging.204609)
Supplement: Supplementary Figures [file aging-15-204609-s001.pdf]

SUPPLEMENTARY FIGURES

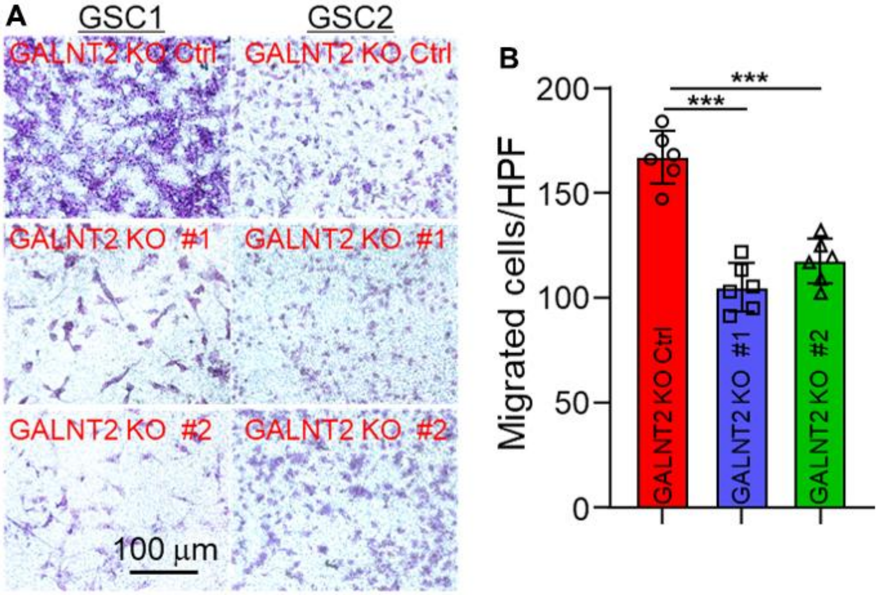

Supplementary Figure 1. GALNT2 ablation impairs GSC migration. (**A**) Representative images of migrated GSCs of indicated group. Scale bar: 100 μm. (**B**) Quantifications of (**A**). *** $p < 0.001$.

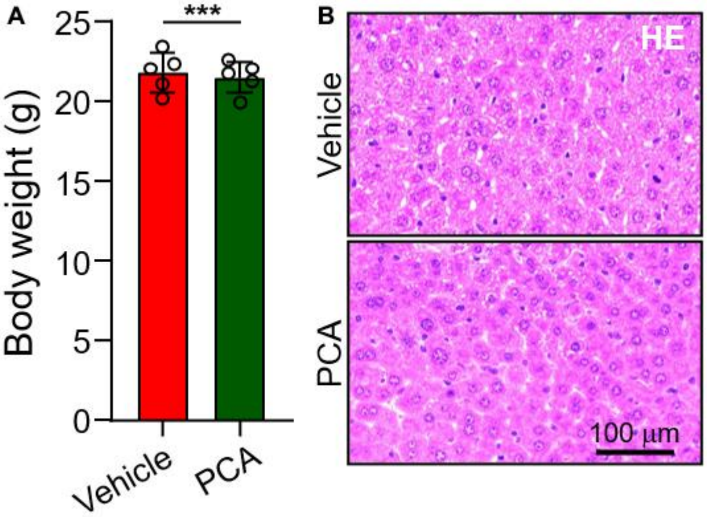

Supplementary Figure 2. PCA did not exert a significant influence on general animal health. (**A**) Body weight of mice treated with vehicle control (1% DMSO) or PCA. (**B**) HE staining of liver of mice treated with vehicle control or PCA.
